# Efficacy of health ecology-based health education on early rehabilitation outcomes in stroke patients

**DOI:** 10.3389/fneur.2025.1659024

**Published:** 2025-10-29

**Authors:** Chengcheng Zhu, Chengshi Zhang, Min Zhang, Liwei Wu, Jia Liu, Xiaoying Liu, Min Shang, Ting Zhang, Yingshu Kuai

**Affiliations:** ^1^Academic Affairs Office, The First Affiliated Hospital of Qiqihar Medical University, Qiqihar, China; ^2^Department of Neurology, The First Affiliated Hospital of Qiqihar Medical University, Qiqihar, China; ^3^Department of Nursing, The First Affiliated Hospital of Qiqihar Medical University, Qiqihar, China; ^4^Department of Rehabilitation, The First Affiliated Hospital of Qiqihar Medical University, Qiqihar, China

**Keywords:** health ecology, health education, nursing, stroke, early rehabilitation

## Abstract

**Background:**

Stroke is a leading global cause of disability and mortality in adults, and early rehabilitation is critical for improving patients’ functional recovery and quality of life; however, conventional rehabilitation models often focus solely on medical interventions and overlook the impact of the patient’s comprehensive ecological environment on recovery, making it necessary to explore more holistic intervention approaches. To investigate the efficacy of health ecology theory-based health education in early stroke rehabilitation and its impacts on neurological/motor/cognitive function, psychological status, and metabolic biomarkers.

**Methods:**

A total of 150 stroke patients during June–October 2024 were randomized into control and intervention groups based on interventions. Outcomes assessed included National Institutes of Health Stroke Scale (NIHSS) for neurological deficit, Fugl-Meyer Assessment (FMA) for motor function, Modified Barthel Index (MBI) for activities of daily living, Montreal Cognitive Assessment (MoCA) for cognition, Hospital Anxiety and Depression Scale (HADS) for psychological status, and metabolic biomarkers glycated hemoglobin (HbA1c) and low-density lipoprotein cholesterol (LDL-C). Structural equation modeling (SEM) was employed to analyze path relationships among metabolic_indices, recovery_effect, and mental_status.

**Results:**

The intervention group demonstrated significantly greater improvement in NIHSS, FMA, MoCA, HbA1c, and LDL-C. Multivariate linear regression models showed moderate-to-substantial explanatory power, particularly for LDL-C and HbA1c (adjusted R^2^ ≈ 30%). Although models for HADS and MBI had lower explanatory power, the intervention effect remained statistically significant (*p* < 0.05). SEM revealed a significant positive path from mental_status to metabolic_indices (*β* = 0.42). The negative path from recovery_effect to metabolic_indices (β = −0.45) signified that better recovery correlated with better metabolic profiles. A significant indirect effect of the intervention on metabolic_indices via improved mental_status was identified (*β* = 1.00 × 0.42).

**Conclusion:**

Health ecology-based health education effectively improves neurological/motor/cognitive function and metabolic control during early stroke rehabilitation. The intervention establishes a “physiological-psychological-social” virtuous cycle through multidimensional effects, providing an innovative framework for collaborative stroke recovery management.

## 1 Introduction

Stroke remains one of the leading global causes of disability and mortality among adults, imposing a substantial burden on patients and their families due to its high incidence and debilitating consequences (1). While modern medicine has achieved significant progress in acute stroke management, reducing mortality rates, the majority of survivors continue to experience varying degrees of functional impairment that severely compromises their quality of life (2). Early rehabilitation is widely acknowledged as a crucial intervention for improving functional outcomes and enhancing the capacity for activities of daily living (ADLs) in stroke patients (3). Nevertheless, conventional rehabilitation models often prioritize medical interventions, overlooking the significant influence of the patient’s complex ecological environment on the recovery trajectory (4).

Health ecology theory emphasizes that individual health results from dynamic interactions among biological, psychological, social, and environmental factors (5). From this perspective, stroke rehabilitation extends beyond clinical treatment and therapeutic exercise; it is profoundly shaped by the patient’s lifestyle, family support system, community environment, and sociocultural context (6). Specifically, a patient’s self-management abilities and health beliefs directly influence adherence to rehabilitation regimens (7). Family financial status, living conditions, caregiver attitudes, and caregiving capacity play pivotal roles in daily assistance, supervision of rehabilitation exercises, and psychological support. The availability of accessible rehabilitation resources and barrier-free environments within the community determines the continuity of rehabilitation efforts and successful societal reintegration (8). Concurrently, sociocultural perceptions and attitudes toward disability can significantly impact rehabilitation motivation and psychological well-being (9). Consequently, delivering health education grounded in health ecology theory, which holistically addresses these interconnected layers of the patient’s ecosystem, holds considerable theoretical and practical significance for optimizing early rehabilitation outcomes and promoting comprehensive recovery after stroke.

## 2 Materials and methods

This study was approved by the Medical Ethics Committee of the First Affiliated Hospital of Qiqihar Medical University (2024-73).

### 2.1 Study population

A total of 150 stroke patients admitted to the Department of Neurology in hospital between June 2024 and October 2024 were enrolled and randomly assigned via a random number table to either a control group (receiving conventional care, *n* = 75) or an intervention group (receiving health ecology theory-based health education, *n* = 75). Random number generation: A statistician independent of the research team used the sample() function in R software (version 4.4.3) to generate a sequence of 150 random numbers (range: 1–150), with even numbers assigned to the control group and odd numbers assigned to the intervention group. The generated random number sequence and corresponding group assignments were sealed in sequentially numbered, opaque envelopes by the statistician. The sample size calculation was performed using the pwr package in R software. Based on an anticipated effect size (d) of 0.67 derived from the literature, a significance level (*α*) of 0.05, and a statistical power of 0.8, the calculated minimum sample size was approximately 35 per group. Accounting for an estimated 20% attrition rate, the required sample size per group was therefore 70/0.8 ≈ 88. Consequently, the enrollment of 150 patients (75 per group) represented a sufficiently powered and statistically robust design to adequately test the intervention effect. Baseline characteristics demonstrated no significant differences between the two groups (Table 1), permitting subsequent comparative analyses.

**Table 1:** Baseline clinical characteristics.

		Control group (*n* = 75)	Intervention group (*n* = 75)	t/χ^2^	*p*
NIHSS		12.34 ± 3.21	12.65 ± 3.15	0.689	0.492
FMA		38.56 ± 8.72	39.02 ± 8.65	0.385	0.701
MBI		56.78 ± 12.34	57.21 ± 12.15	0.264	0.792
MoCA		22.34 ± 3.56	22.56 ± 3.48	0.412	0.681
HADS		8.76 ± 2.34	8.92 ± 2.28	0.485	0.628
HbA1c (%)		6.21 ± 0.56	6.18 ± 0.54	0.392	0.696
LDL-C (mmol/L)		3.21 ± 0.78	3.18 ± 0.75	0.267	0.79
Hct		0.42 ± 0.05	0.43 ± 0.04	1.235	0.219
2-h postprandial blood glucose (mmol/L)		7.89 ± 1.23	8.02 ± 1.18	0.765	0.446
PT (seconds)		12.34 ± 1.21	12.45 ± 1.18	0.682	0.497
APTT (seconds)		28.76 ± 3.45	28.92 ± 3.38	0.315	0.753
D-D (mg/L)		0.34 ± 0.12	0.36 ± 0.11	1.025	0.307
Fasting blood glucose (mmol/L)		5.67 ± 0.89	5.72 ± 0.85	0.398	0.691
CRP (mg/L)		8.76 ± 2.34	8.91 ± 2.28	0.475	0.635
Hcy (μmol/L)		12.34 ± 2.56	12.51 ± 2.48	0.458	0.648
Disease duration (days)		3.45 ± 1.23	3.58 ± 1.19	0.762	0.448
Age (years)		65.43 ± 8.76	66.02 ± 8.65	0.489	0.625
Gender	Male	36 (48)	41 (55.67)	0.667	0.414
	Female	39 (52)	34 (45.33)		

NIHSS, National Institutes of Health Stroke Scale; FMA, Fugl-Meyer Assessment; MBI, Modified Barthel Index; MoCA, Montreal Cognitive Assessment; HADS, Hospital Anxiety and Depression Scale; HbA1c, glycosylated Hemoglobin; LDL-C, low-density lipoprotein cholesterol; Hct, hematocrit; PT, prothrombin time; APTT, activated partial thromboplastin time; D-D, D-dimer; CRP, C-reactive protein; Hcy, homocysteine.

Inclusion criteria comprised: (1) Diagnosis of ischemic stroke (including cerebral infarction, cerebral embolism) or hemorrhagic stroke (including intracerebral hemorrhage, subarachnoid hemorrhage) confirmed by neuroimaging [transcranial computed tomography (CT) or magnetic resonance imaging (MRI)] according to World Stroke Organization (WSO) diagnostic criteria (10); (2) Stable vital signs; (3) Age between 18 and 80 years; (4) Recruited at the early rehabilitation phase of stroke (within 2 weeks after the onset of stroke), and conscious; (5) First-episode or recurrent stroke patients are both included; for recurrent stroke patients, additional conditions must be met: the current stroke episode conforms to the above diagnostic criteria (Item 1) and vital sign stability criteria (Item 2), and the interval from the previous stroke onset is ≥ 6 months (to avoid the residual functional impairment of the previous stroke interfering with the evaluation of the current intervention effect); (6) Provision of written informed consent by the patients or their legally authorized representatives, residence within the geographical area accessible to the research team for regular follow-up visits and guidance, and availability of a family or social support system capable of assisting the patient with rehabilitation exercises during daily living.

Exclusion criteria included: (1) Severe comorbidities (e.g., significant dysfunction of major organs such as the heart, lung, liver, or kidney) precluding tolerance of the physical or psychological demands of early rehabilitation training and health education, or likely to confound study outcomes; (2) Presence of psychiatric disorders or cognitive impairment (MoCA score ≤ 17); (3) Critically ill status or a life expectancy estimated by the treating physician to be less than 3 months; (4) Other factors potentially interfering with study participation (e.g., history of significant neurological conditions unrelated to stroke).

### 2.2 Nursing interventions

Both groups received interventions for a duration of 6 months. Nurses assigned to the intervention group underwent standardized training and competency assessment prior to participation in the study.

#### 2.2.1 Control group

Patients received conventional stroke nursing care. For acute-phase patients, continuous monitoring of vital signs and neurological status was performed, alongside vigilant observation for complications. Proper positioning was rigorously maintained: in the supine position, a pillow supported the head, with soft pads placed under the shoulders and hips to prevent scapular retraction and posterior pelvic tilt. The affected upper limb was positioned in extension, palm supinated and fingers abducted, resting on a pillow. The affected lower limb was slightly flexed at the knee, supported by a pad to prevent external rotation. Patients were repositioned every 2 h to minimize prolonged pressure on any single area and prevent pressure ulcers. Repositioning maneuvers were performed gently, ensuring the head, trunk, and limbs remained aligned to avoid torsion. When clinically stable, the head of the bed was elevated 30°–45°, gradually progressing to a seated position. Initial sitting periods were brief (5–10 min), carefully monitoring for dizziness or palpitations, and duration was progressively increased. Airway management included maintaining patency by promptly clearing oral and respiratory secretions. Comatose patients were positioned laterally to prevent tongue obstruction and aspiration. Suctioning was performed as needed, preceded by oxygenation, using gentle technique to avoid mucosal trauma. Patients were instructed in deep breathing and effective cough exercises every 2–3 h. Nebulization therapy was administered for patients with tenacious sputum to facilitate expectoration, complemented by regular chest percussion. Early ambulation was encouraged; bed-bound patients received regular repositioning and percussion, with environmental measures (e.g., adequate room ventilation) implemented to prevent cross-infection. Within 2–3 days post-stroke, if conscious and without dysphagia, patients commenced a liquid diet, advancing progressively to soft solids and a regular diet as tolerated. Dietary recommendations emphasized low sodium, low fat, high protein, and high vitamin content, avoiding spicy or irritating foods. Patients with dysphagia underwent swallowing assessments, with dietary texture modified accordingly (e.g., pureed foods, avoiding dry or sticky consistencies). Feeding occurred in an upright or semi-Fowler’s position with slight neck flexion, and patients were instructed to eat slowly and avoid talking to prevent aspiration. Enteral nutrition via nasogastric tube was provided for patients unable to eat orally, ensuring correct tube placement, appropriate formula temperature, volumes ≤ 200 mL per feeding, and intervals ≥ 2 h. Nutritional status was monitored regularly, with dietary plans adjusted as needed. Urinary function was observed, and interventions for urinary retention included suprapubic heat application and bladder massage, with catheterization performed if necessary. Indwelling catheters were managed to ensure patency and prevent urinary tract infections through regular catheter and bag changes. Patients were encouraged to consume fiber-rich foods such as vegetables and fruits and establish regular bowel habits. Constipation was managed with glycerin suppositories, oral laxatives, or enemas as required (11–15).

For convalescent-phase patients, care transitioned to rehabilitation-focused nursing, encompassing: (1) Rehabilitation training: Developing individualized physiotherapy plans based on the degree of limb paresis; conducting speech-language assessments and implementing tailored therapy for aphasia; continuing swallowing therapy for residual dysphagia. (2) Psychological support: Identifying psychological issues through active communication and behavioral observation; providing empathetic support, encouraging expression of feelings and needs; educating patients about stroke and sharing successful rehabilitation cases to bolster confidence. (3) Safety measures: Maintaining dry, clutter-free floors; ensuring supervised ambulation (family or staff) with assistive devices as needed; advising bed rest for patients experiencing dizziness or fatigue; regulating temperature (<50 °C) and using protective covers for heating devices such as hot water bottles and thermoses to prevent burns. (4) Discharge planning: Providing detailed instructions on post-discharge medications (names, actions, dosages, side effects, precautions), lifestyle modifications, and safe use of rehabilitation equipment at home for both patients and caregivers.

#### 2.2.2 Intervention group

In addition to the conventional care described above, patients received structured health education grounded in health ecology theory (16–18). This multifaceted program spanned four interconnected levels:

##### 2.2.2.1 Individual level

Providing detailed education on stroke pathophysiology, clinical manifestations, treatment options, and the rehabilitation process. Utilizing illustrated brochures and educational videos, patients were taught to differentiate ischemic from hemorrhagic stroke and recognize critical warning signs (e.g., facial asymmetry, slurred speech, hemiparesis) emphasizing the urgency of seeking immediate medical attention. Explanations of therapeutic approaches (medications, surgery, rehabilitation) aimed to enhance disease understanding and alleviate fear/anxiety related to the unknown. Nurses collaborated with patients to develop personalized dietary modifications and feasible exercise plans, and trained patients/families in basic self-monitoring techniques (blood pressure, blood glucose, heart rate).

##### 2.2.2.2 Family level

Actively fostering a robust family support system. Families were encouraged to provide consistent emotional support (care, companionship, encouragement) and practical assistance with ADLs. Crucially, families received guidance and training to effectively support and supervise the patient’s prescribed rehabilitation exercises within the home environment.

##### 2.2.2.3 Community level

Integrating local rehabilitation resources to enhance accessibility. This involved liaising with community health centers to utilize their rehabilitation equipment and therapist services, facilitating convenient access for stroke patients.

##### 2.2.2.4 Societal level

Implementing broader societal education and advocacy. Nurses advocated to government entities regarding the public health importance of stroke prevention and management, seeking supportive policies. Mass media platforms (television, radio, print, online) were leveraged to disseminate stroke prevention knowledge widely through public service announcements and health programs. Stroke education was embedded within national health literacy campaigns, promoting healthy lifestyles and disease prevention awareness. Efforts were also directed toward societal education to foster greater understanding, acceptance, and support for stroke survivors, reducing stigma and creating a more enabling social environment for recovery.

### 2.3 Outcome measures

Blinding process: Considering the nature of nursing intervention studies (patients and nurses implementing the intervention can easily identify group assignments due to differences in care content), complete double-blinding (participants + intervention implementers) was not feasible. To minimize detection bias and performance bias, the following partial blinding measures were adopted: Blinding of outcome assessors: Neurologists, rehabilitation therapists, and laboratory technicians responsible for outcome measurement (NIHSS, FMA, MoCA, MBI, HADS assessment, and HbA1c/LDL-C detection) were kept unaware of patient group assignments. They only received patient ID numbers and no information about whether the patient received conventional care or the intervention. Blinding of data collectors and analysts: Research assistants who collected clinical data (e.g., disease duration, concomitant medications) used a standardized data collection form with only patient ID numbers; the statistician who conducted data analysis (multivariate regression, SEM) used de-identified data (group labels were replaced with “Group A” and “Group B” during analysis) to avoid subjective bias.

A comprehensive battery of validated instruments and laboratory assays was employed to assess patient outcomes. Neurological deficit severity was evaluated using the National Institutes of Health Stroke Scale (NIHSS) (19), which quantified impairments across domains including level of consciousness, gaze, visual fields, facial palsy, motor function in limbs, sensation, and language. The NIHSS yielded a total score ranging from 0 to 42, with higher scores indicating greater neurological impairment.

Motor function recovery was assessed with the Fugl-Meyer Assessment (FMA) (20). This scale provided separate scores for the upper extremity (maximum 66 points) and lower extremity (maximum 34 points), culminating in a total motor score out of 100, where higher scores reflected better motor performance.

Functional independence in ADLs was measured using the Modified Barthel Index (MBI) (21). This index evaluated performance across 10 essential activities, such as feeding, bathing, dressing, toileting, and ambulation, generating a total score between 0 and 100, with increased scores denoting greater self-care ability.

Cognitive function was screened with the Montreal Cognitive Assessment (MoCA) (22). This instrument assessed multiple cognitive domains, including attention, memory, language, and visuospatial abilities, providing a total score out of 30. A score of ≤26 was generally indicative of cognitive impairment, with adjustments applied based on the patient’s educational level.

Emotional status was evaluated using the Hospital Anxiety and Depression Scale (HADS) (23), which generated separate subscale scores for anxiety and depression, each ranging from 0 to 21.

Laboratory biomarkers were systematically monitored. Glycated hemoglobin (HbA1c) and homocysteine (Hcy) levels were quantified using high-performance liquid chromatography (HPLC). D-dimer (D-D) and C-reactive protein (CRP) concentrations were determined via immunoturbidimetric assays. Blood glucose levels (fasting and 2-h postprandial) were measured using the glucose oxidase method. Hematocrit (Hct) was analyzed using standard hematological techniques. Additionally, clinically relevant data including low-density lipoprotein cholesterol (LDL-C), prothrombin time (PT), and activated partial thromboplastin time (APTT) were collected from patient records.

### 2.4 Model construction

Guided by health ecology theory, a multi-tiered influence model encompassing “individual, family, community, and society” levels was conceptualized. Structural equation modeling (SEM) was implemented using the lavaan package within the R statistical environment. Model parameters were estimated using robust maximum likelihood estimation (MLR) to accommodate potential non-normality in the data. Model fit was rigorously evaluated employing established indices, including the normed chi-square (χ^2^/df), root mean square error of approximation (RMSEA), and comparative fit index (CFI). The statistical significance of mediating effects within the model was assessed using the Bootstrap method with 5,000 resamples to generate bias-corrected confidence intervals.

The theoretical framework dictated the specification of path relationships involving four exogenous latent variables and one endogenous latent variable. Each latent variable was operationally defined by its respective set of observed indicators (XX indicators per latent construct). The internal consistency reliability of the scales constituting these indicators was confirmed, with all Cronbach’s *α* coefficients exceeding 0.7. Additionally, radar charts were generated using the fmsb package in R to provide a visual comparison of profile patterns between groups. These charts were constructed using optimal and minimal reference boundaries derived from the data, with distinct color schemes representing the two study groups and clear labels applied for interpretability.

### 2.5 Statistical analysis

All statistical analyses were performed using SPSS software (version 27.0) and the R statistical computing environment (version 4.4.3). Continuous variables demonstrating approximate normal distribution were presented as mean ± standard deviation ($\bar{x}$ ± s). Categorical variables were summarized as n (%). Between-group comparisons were conducted using independent samples *t*-tests and chi-square (χ^2^) tests. To identify significant independent predictors among the variables examined, multivariate binary logistic regression analysis was implemented. Data visualization was accomplished using the ggplot2 package within R. A *p*-value of less than 0.05 was considered statistically significant.

## 3 Results

### 3.1 Comparison of post-intervention clinical outcomes

**Figure 1:**
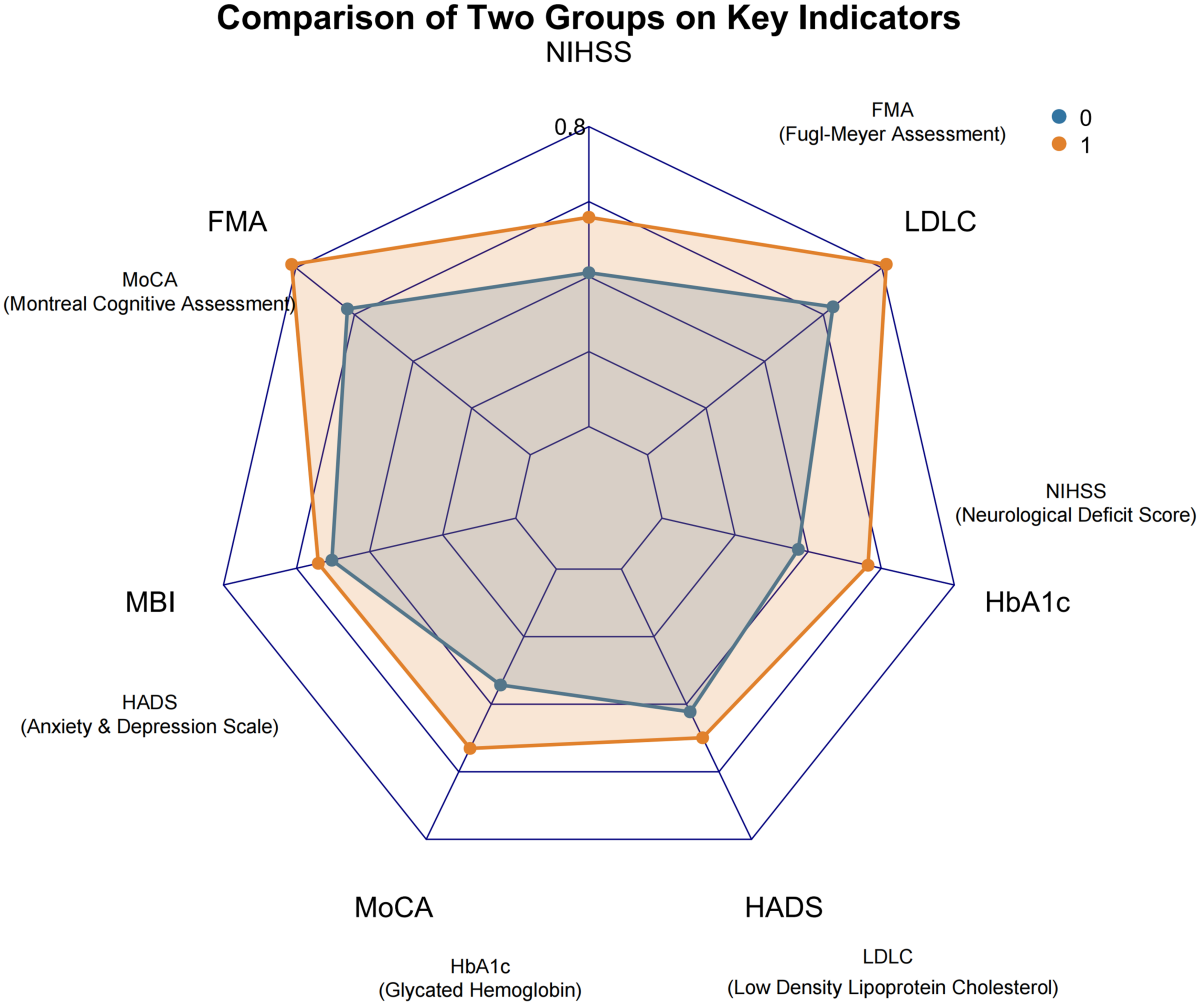
Radar chart visualization of key outcome measures between control and intervention groups.

Comparative analysis revealed statistically significant differences between the control and intervention groups across key clinical and functional domains following the intervention period. Specifically, significant disparities were observed in the scores of NIHSS, FMA, MBI, MoCA, and HADS. Among the serological biomarkers assessed, HbA1c and LDL-C levels also demonstrated significant intergroup differences. The contrasting profiles between the two groups for these significantly divergent parameters were further elucidated through a radar chart visualization, highlighting distinct patterns of outcome attainment. All reported differences achieved statistical significance at *p* < 0.05. Comprehensive comparative data are presented in Table 2, with the corresponding radar chart illustrated in Figure 1.

**Table 2:** Comparative analysis of post-intervention clinical outcomes between groups.

Indicator	Control group (*n* = 75)	Intervention group (*n* = 75)	*t*	*p*
Hct	0.35 ± 0.04	0.36 ± 0.03	1.286	0.201
2-h postprandial blood glucose (mmol/L)	7.25 ± 1.15	7.32 ± 1.10	0.421	0.674
PT (seconds)	11.98 ± 1.15	12.05 ± 1.12	0.389	0.698
APTT (seconds)	27.52 ± 3.20	27.68 ± 3.15	0.312	0.755
D-D (mg/L)	0.28 ± 0.10	0.30 ± 0.09	1.254	0.212
Fasting blood glucose (mmol/L)	5.02 ± 0.82	5.08 ± 0.78	0.456	0.649
CRP (mg/L)	7.25 ± 2.10	7.38 ± 2.05	0.381	0.704
Hcy (μmol/L)	11.21 ± 2.30	11.36 ± 2.25	0.405	0.686
NIHSS	20.45 ± 11.19	12.69 ± 7.08	5.076	<0.001
FMA	51.49 ± 27.49	67.19 ± 17.68	−4.159	<0.001
MBI	56.99 ± 16.85	67.56 ± 17.13	−3.811	<0.001
MoCA	12.87 ± 7.32	19.91 ± 6.09	−6.404	<0.001
HADS	9.91 ± 5.27	7.89 ± 4.57	2.499	0.014
HbA1c (%)	8.27 ± 1.72	6.36 ± 1.37	7.534	<0.001
LDL-C (mmol/L)	2.47 ± 0.65	1.74 ± 0.45	7.96	<0.001

Hct, hematocrit; PT, prothrombin time; APTT, activated partial thromboplastin time; D-D, D-dimer; CRP, C-reactive protein; Hcy, homocysteine; NIHSS, National Institutes of Health Stroke Scale; FMA, Fugl-Meyer Assessment; MBI, Modified Barthel Index; MoCA, Montreal Cognitive Assessment; HADS, Hospital Anxiety and Depression Scale; HbA1c, glycosylated Hemoglobin; LDL-C, low-density lipoprotein cholesterol.

### 3.2 Impact of intervention modality on patient outcomes

To isolate the independent effect of the intervention modality on specific patient outcomes, multivariate binary logistic regression analysis was performed, with the control group coded as 0 and the intervention group coded as 1. The analysis revealed that the assigned intervention modality demonstrated significant associations with several key outcome measures. Specifically, scores on NIHSS and MoCA, and levels of HbA1c and LDL-C were significantly influenced by the intervention group assignment, as presented in Table 3.

**Table 3:** Multivariate binary logistic regression analysis of intervention effects on key outcomes.

	B	SE	W	*p*	OR	OR 95% CI	
						Lower	Upper
NIHSS	−0.089	0.032	7.749	0.005	0.915	0.86	0.974
FMA	0.022	0.013	3.083	0.079	1.023	0.997	1.048
MBI	0.006	0.016	0.152	0.696	1.006	0.975	1.038
MoCA	0.144	0.045	10.336	0.001	1.155	1.058	1.260
HADS	−0.053	0.059	0.813	0.367	0.948	0.845	1.064
HbA1c	−0.789	0.200	15.620	< 0.001	0.454	0.307	0.672
LDL-C	−1.776	0.539	10.857	0.001	0.169	0.059	0.487
Constant	7.090	2.487	8.126	0.004	1199.756		

NIHSS, National Institutes of Health Stroke Scale; FMA, Fugl-Meyer Assessment; MBI, Modified Barthel Index; MoCA, Montreal Cognitive Assessment; HADS, Hospital Anxiety and Depression Scale; HbA1c, glycosylated Hemoglobin; LDL-C, low-density lipoprotein cholesterol; SE, standard error; OR, odds ratio; CI, confidence interval.

- LDL-C (R2 = 29.50%): The intervention explained nearly 30% of the variance in LDL-C levels—this is clinically meaningful because LDL-C is a modifiable risk factor for stroke recurrence. A 29.5% explanation of LDL-C variance indicates the intervention is a key driver of lipid improvement, beyond other factors (e.g., medication, diet).
- HbA1c (R2 = 27.23%): The intervention accounted for ~27% of HbA1c variance. Since HbA1c reflects 3-month glycemic control, this high R2 confirms the intervention’s sustained impact on metabolic health—superior to conventional care’s typical 10–15% explanatory power for glycemic outcomes (24).
- MoCA (R2 = 21.17%): The intervention explained ~21% of cognitive function variance. Given that post-stroke cognitive impairment is multifactorial (e.g., brain lesions, aging), a 21% contribution from the intervention highlights its role as a modifiable factor for cognitive recovery.
- NIHSS (R2 = 14.25%) and FMA (R2 = 9.86%): While these R2 values are lower, they remain clinically relevant—neurological and motor recovery are influenced by lesion location/size (non-modifiable factors), so a 9.8–14.25% contribution from the intervention (a modifiable factor) is substantial for clinical practice.

### 3.3 SEM path analysis revealing latent relationships among variables

The SEM analysis elucidated the latent relationships among key constructs. The model incorporated three primary latent variables: (1) metabolic_indices, representing the underlying metabolic construct, reflected by the observed indicators HbA1c and LDL-C; (2) recovery_effect, representing the latent construct of rehabilitation outcomes, reflected by the observed indicators FMA, MBI, MoCA, and NIHSS; and (3) mental_status, representing the latent construct of psychological well-being, associated with the HADS. Factor loadings quantified the strength of the relationship between each latent variable and its observed indicators. Significant standardized loadings were observed for metabolic_indices→HbA1c_std (loading = 0.53) and metabolic_indices → LDL-C_std (loading = 0.54), indicating that the metabolic latent construct strongly explained variance in both biomarkers. Similarly, recovery_effect → FMA_std demonstrated a loading of 0.36, reflecting the contribution of motor function to the overall recovery construct.

**Figure 2:**
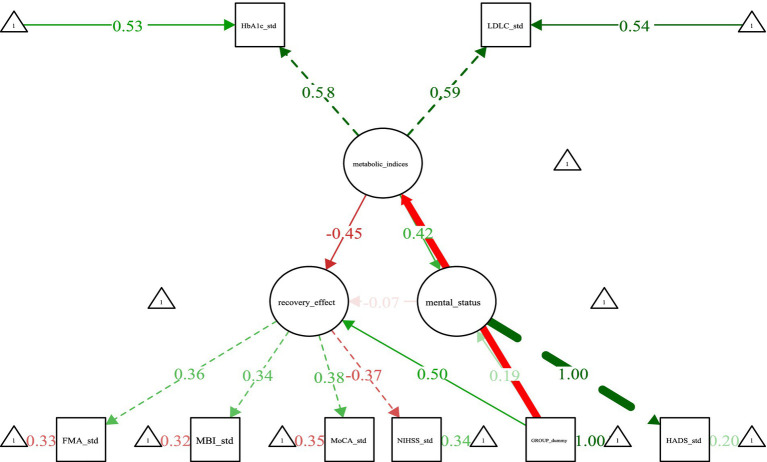
Standardized path diagram of the structural equation model (SEM). std. denotes standardized values; paths significant at *p* < 0.05 are shown.

Path coefficients revealed the directional relationships between latent variables. Mental_status → metabolic_indices (*β* = 0.42, green) indicated a significant positive influence, suggesting that better psychological status (e.g., less anxiety/depression) is associated with more favorable metabolic profiles (e.g., lower HbA1c, lower LDL-C). Recovery_effect → metabolic_indices (β = −0.45, red) indicated a significant negative influence. Crucially, as higher NIHSS scores represent worse neurological function (a negative indicator within the recovery_effect construct), this negative path coefficient translates to a positive real-world association: better overall rehabilitation outcomes (lower NIHSS, higher FMA/MBI/MoCA) are associated with better metabolic profiles. Mental_status → recovery_effect (*β* = −0.07, light red) demonstrated a weak, non-significant predictive utility of psychological status on the recovery outcome construct within this model. Furthermore, the model indicated that the intervention modality exerted a strong influence on mental_status (β = 1.00,). Given the established path mental_status → metabolic_indices (β = 0.42), this suggests a significant indirect effect of the intervention on metabolic indices via improvement in psychological status. The analysis also confirmed the positive association between recovery_effect and metabolic_indices, as interpreted from the significant negative path coefficient considering the NIHSS indicator direction. The standardized path diagram illustrating these relationships is presented in Figure 2.

### 3.4 Multivariate linear regression model results

The multivariate linear regression analysis demonstrated that the intervention modality exerted significant independent effects on neurological function, motor function, cognitive function, and metabolic biomarkers. The adjusted R-squared (R^2^) values indicated moderate to substantial explanatory power for these models, notably approaching nearly 30% for both LDL-C and HbA1c. This robust explanatory capacity strongly suggests that the intervention modality was a significant determinant of these specific outcomes. Although the models for the HADS and MBI yielded lower explanatory power (adjusted R^2^ = 3.4 and 8.3%, respectively), the corresponding *p*-values for the intervention effect attained statistical significance. This indicates that, despite the models explaining a smaller proportion of the variance, the intervention modality likely exerted a statistically significant, albeit modest, impact on psychological status and ADLs within the context of this analysis (Table 4).

**Table 4:** Multivariate linear regression analysis examining intervention effects on key outcomes.

Indicator	R^2^	*p*	Significance and interpretation
NIHSS	0.1425	1.14 × 10^−6^ (<0.001)	Intervention explained 14.25% of NIHSS variance, indicating significant association with neurological deficit severity
FMA	0.0986	5.40 × 10^−5^ (<0.001)	Intervention explained 9.86% of FMA variance, demonstrating significant effect on motor function
MBI	0.0832	0.0002 (<0.001)	Intervention explained 8.32% of MBI variance, showing significant correlation with ADLs
MoCA	0.2117	1.89 × 10^−9^ (<0.001)	Intervention explained 21.17% of MoCA variance, reflecting strong association with cognitive function
HADS	0.0340	0.0136 (<0.05)	Intervention explained 3.40% of HADS variance, indicating significant link to anxiety/depression status
HbA1c	0.2723	4.52 × 10^−12^ (<0.001)	Intervention explained 27.23% of HbA1c variance, confirming strong impact on glycemic control
LDL-C	0.295	4.16 × 10^−13^ (<0.001)	Intervention explained 29.50% of LDL-C variance, evidencing highly significant influence on lipid profile

NIHSS, National Institutes of Health Stroke Scale; FMA, Fugl-Meyer Assessment; MBI, Modified Barthel Index; MoCA, Montreal Cognitive Assessment; HADS, Hospital Anxiety and Depression Scale; HbA1c, glycosylated Hemoglobin; LDL-C, low-density lipoprotein cholesterol; ADLs, activities of daily living.

## 4 Discussion

Stroke persists as the second leading cause of global mortality and the third leading contributor to death and disability worldwide. The past two decades (1990–2019) witnessed a concerning escalation, with a 70% increase in absolute stroke incidence, an 85% rise in stroke prevalence, a 43% increase in stroke-related deaths, and a 32% surge in disability-adjusted life years (DALYs) (24). Our approach aims to address the inherent limitations of individual-centric models by enhancing systemic comprehensiveness and integration, thereby holding significant potential for clinical practice and broader societal benefit.

Utilizing SEM, we constructed a network of latent variable relationships encompassing metabolic indices, recovery outcomes, and psychological status. Within this model, metabolic_indices emerged as a robust core indicator reflecting overall metabolic health. The recovery_effect construct highlighted motor function (as measured by the FMA) as its most salient contributing factor, aligning with the established clinical priority of motor recovery as a primary early rehabilitation goal (25). The close association between mental_status and HADS scores underscores the representative nature of anxiety and depression within the psychological well-being construct. A particularly noteworthy finding was the significant indirect pathway identified: the intervention modality exerted a positive influence on mental_status, which subsequently predicted improved metabolic_indices. This pathway validates the core hypothesis derived from health ecology theory that multi-level interventions can optimize metabolic management, at least partly, through enhancing psychological well-being. Furthermore, the identified positive association between recovery_effect and metabolic_indices suggests a potential bidirectional, synergistic relationship. Optimal metabolic control likely provides a physiological foundation conducive to effective rehabilitation, while active engagement in rehabilitation activities may reciprocally promote further metabolic improvements. These results are consistent with QUAN L’s research on neurology (26).

Results from the multivariate linear regression analysis confirmed significant independent effects of the intervention modality on a broad spectrum of outcomes, including neurological function (NIHSS), motor function (FMA), cognitive function (MoCA), metabolic biomarkers (HbA1c, LDL-C), psychological status (HADS), and ADLs (MBI). The substantial explanatory power of the models, particularly for LDL-C and HbA1c (adjusted R^2^ approaching 30%), indicates that the health ecology-based intervention accounted for approximately 30% of the variance in these key glucose-lipid metabolism parameters. This strong association is plausibly linked to the personalized dietary guidance, tailored exercise plans, and structured family supervision mechanisms embedded within the intervention. Neurological and cognitive outcomes demonstrated moderate explanatory power (adjusted R^2^ between 14.25 and 21.17%), suggesting that the intervention’s promotion of neuroplasticity may be mediated through multi-faceted stimulation strategies, such as community-based cognitive training and family interaction activities. From a clinical perspective, the high explanatory power for metabolic biomarkers is highly significant, given that effective glycemic and lipid control substantially mitigates the risk of stroke recurrence. This finding provides empirical support for an intervention strategy focused on achieving synergistic improvements in metabolic and neurological function through multi-dimensional health education, showcasing the distinct advantage of the health ecology model in managing chronic conditions like stroke.

In summary, the multi-layered health education model, conceptualized through the lens of health ecology theory, demonstrates significant efficacy in early stroke rehabilitation. It facilitates improvements not only in neurological, motor, and cognitive functions but also optimizes glucose-lipid metabolism parameters through the modulation of psychological status, fostering a positive interplay across physiological, psychological, and social dimensions. Nevertheless, several limitations warrant consideration. Firstly, recruitment from a single medical center introduces potential selection bias and limits the generalizability of the findings. Secondly, the observation period was confined to the early rehabilitation phase (within 2 weeks post-stroke), lacking long-term follow-up data on outcomes such as ADL capacity at 6 months or beyond. Thirdly, within the SEM framework, the factor loading for certain observed indicators (e.g., FMA on the recovery_effect construct, loading = 0.36) was relatively modest, suggesting potential benefit from incorporating additional dimensions (e.g., pain severity, sleep quality) to enhance model fit and comprehensiveness in future iterations. Finally, the standardization fidelity and implementation consistency of the complex intervention were not formally quantified, which may impact the reproducibility of the results. Future research should prioritize larger-scale, multi-center randomized controlled trials with extended follow-up periods. Refining the relative weighting of interventions across the different ecological levels and integrating qualitative methodologies to explore patients’ subjective experiences will be crucial for generating more robust evidence and further optimizing health ecology-based intervention protocols for stroke rehabilitation.

## Data Availability

The original contributions presented in the study are included in the article/supplementary material, further inquiries can be directed to the corresponding author.
